# Progressive Transition From Supervised to Unsupervised Robot-Assisted Therapy After Stroke: Protocol for a Single-Group, Interventional Feasibility Study

**DOI:** 10.2196/48485

**Published:** 2023-11-09

**Authors:** Giada Devittori, Raffaele Ranzani, Daria Dinacci, Davide Romiti, Antonella Califfi, Claudio Petrillo, Paolo Rossi, Roger Gassert, Olivier Lambercy

**Affiliations:** 1 Rehabilitation Engineering Laboratory Swiss Federal Institute of Technology Zürich Zurich Switzerland; 2 Clinica Hildebrand Centro di Riabilitazione Brissago Brissago Switzerland; 3 Future Health Technologies Singapore-ETH Centre Campus for Research Excellence and Technological Enterprise (CREATE) Singapore Singapore

**Keywords:** technology-assisted rehabilitation, unsupervised therapy, self-directed therapy, stroke, neurorehabilitation, neurocognitive rehabilitation, robot-assisted therapy, rehabilitation technology, robot.

## Abstract

**Background:**

Increasing the dose of therapy delivered to patients with stroke may improve functional outcomes and quality of life. Unsupervised technology-assisted rehabilitation is a promising way to increase the dose of therapy without dramatically increasing the burden on the health care system. Despite the many existing technologies for unsupervised rehabilitation, active rehabilitation robots have rarely been tested in a fully unsupervised way. Furthermore, the outcomes of unsupervised technology-assisted therapy (eg, feasibility, acceptance, and increase in therapy dose) vary widely. This might be due to the use of different technologies as well as to the broad range of methods applied to teach the patients how to independently train with a technology.

**Objective:**

This paper describes the study design of a clinical study investigating the feasibility of unsupervised therapy with an active robot and of a systematic approach for the progressive transition from supervised to unsupervised use of a rehabilitation technology in a clinical setting. The effect of unsupervised therapy on achievable therapy dose, user experience in this therapy setting, and the usability of the rehabilitation technology are also evaluated.

**Methods:**

Participants of the clinical study are inpatients of a rehabilitation clinic with subacute stroke undergoing a 4-week intervention where they train with a hand rehabilitation robot. The first week of the intervention is supervised by a therapist, who teaches participants how to interact and train with the device. The second week consists of minimally supervised therapy, where the therapist is present but intervenes only if needed as participants exercise with the device. If the participants properly learn how to train with the device, they proceed to the unsupervised phase and train without any supervision during the third and fourth weeks. Throughout the duration of the study, data on feasibility and therapy dose (ie, duration and repetitions) are collected. Usability and user experience are evaluated at the end of the second (ie, minimally supervised) and fourth (ie, unsupervised) weeks, allowing us to investigate the effect of therapist absence.

**Results:**

As of April 2023, 13 patients were recruited and completed the protocol, with no reported adverse events.

**Conclusions:**

This study will inform on the feasibility of fully unsupervised rehabilitation with an active rehabilitation robot in a clinical setting and its effect on therapy dose. Furthermore, if successful, the proposed systematic approach for a progressive transition from supervised to unsupervised technology-assisted rehabilitation could serve as a benchmark to allow for easier comparisons between different technologies. This approach could also be extended to the application of such technologies in the home environment, as the supervised and minimally supervised sessions could be performed in the clinic, followed by unsupervised therapy at home after discharge.

**Trial Registration:**

ClinicalTrials.gov NCT04388891; https://clinicaltrials.gov/study/NCT04388891

**International Registered Report Identifier (IRRID):**

DERR1-10.2196/48485

## Introduction

Stroke survivors often do not entirely recover upper limb function [1], which severely impacts independence and quality of life as remaining upper limb impairments may limit their ability to perform activities of daily living. Increasing evidence shows that therapy dose is a key factor contributing to sensorimotor recovery and that administering a higher dose of upper limb therapy to people after stroke could improve their functional outcome even long after stroke [2,3].

However, due to factors such as unfavorable therapist to patient ratios [4], high rehabilitation-related costs, and the overall frailty of health care systems (as highlighted, for example, by the COVID-19 pandemic [5,6]), increasing the dose of high-quality therapy for both inpatients and outpatients is challenging as long as it relies on hospital visits and supervised therapy.

Unsupervised technology-assisted rehabilitation, defined here as patients training with rehabilitation technologies without any supervision by an external person, is a promising solution to increase therapy dose without dramatically weighing on the health care system [7]. Relying on novel digital approaches, such as wearable sensors or robotic tools, offers new ways to promote motivation and engagement for specific therapy exercises. It promises to go beyond the conventional set of exercises prescribed for patients to train independently, which may lead to low adherence [8,9]. A range of upper limb rehabilitation technologies have been tested in unsupervised settings, such as the home environment, with varying results regarding acceptance, satisfaction, success, and amount of use [10-15]. Besides the use of different technologies, these outcomes may also be influenced by the different methods applied to teach the patients how to use a rehabilitation technology in an independent way. A key step for the successful and safe use of a rehabilitation technology without supervision is the progressive transition toward unsupervised use, where users first learn how to correctly use a technology. This becomes especially critical in the case of active technologies, such as rehabilitation robotics, whose use without any external supervision has rarely been reported.

Unfortunately, most existing studies only report little information on these methodological steps or use very different protocols (eg, from multiple training sessions in supervised settings [11] to a single explanation session directly at home [12]). Similarly, there is a lack of agreement on objective measures to evaluate and document the feasibility and success of unsupervised training.

In this protocol paper, we report on the study design of a single-group, interventional clinical study to test the feasibility of fully unsupervised therapy with an active hand rehabilitation robot in patients with subacute stroke. More specifically, we describe in detail a study protocol with the following aims: (1) to evaluate the feasibility of unsupervised therapy assisted by an active rehabilitation robot and a standardized approach for the progressive transition from supervised to unsupervised use based on a set of well-defined and objective criteria, (2) to investigate the effect of unsupervised therapy on achievable therapy dose during rehabilitation, and (3) to evaluate user experience as well as the usability of the rehabilitation technology.

The secondary objective of this study is to identify patient-related parameters (eg, age, cognitive abilities, level of impairment) that might influence the ability of a user to transition to unsupervised therapy and the dose of self-administered therapy.

## Methods

### Study Population and Recruitment

For this study, we aim to recruit patients in the subacute phase after stroke that are currently undergoing rehabilitation (inpatients). To be eligible for the study, patients must fulfill all the following inclusion criteria: (1) be between 18 and 90 years old, (2) be within 6 weeks from stroke onset, (3) have a prestroke Modified Rankin Score [16] ≤1, (4) have a National Institutes of Health Stroke Scale [17] score ≥1 in at least one of the items concerning motor or sensory function and ataxia, and (5) have provided informed consent as documented by a signature.

Patients are excluded if one of the following exclusion criteria is present: (1) moderate to severe aphasia (Goodglass-Kaplan scale [18] <3), (2) moderate to severe cognitive deficits (levels of cognitive functioning-revised [19] <8), (3) functional impairment of the upper limb due to other pathologies, (4) severe pain in the affected arm (visual analogue scale [VAS] for pain ≥5), (5) other pathologies that may interfere with the study, (6) pacemakers and other active implants, and (7) a modified Ashworth Scale [20] >2 for one or more of the following muscles: shoulder adductors, forearm pronator and supinator, and flexors and extensors of the elbow, wrist, and fingers.

The study is recruiting inpatients with stroke from the Clinica Hildebrand Centro di Riabilitazione Brissago, a rehabilitation clinic in Switzerland where the rehabilitation technology used in the study is available. One of the medical doctors of the rehabilitation clinic involved in the study proposes to patients potentially meeting the eligibility criteria to participate in the study, explains the protocol, and obtains informed consent from them. Compensation is not provided.

### The ReHapticKnob

**Figure 1:**
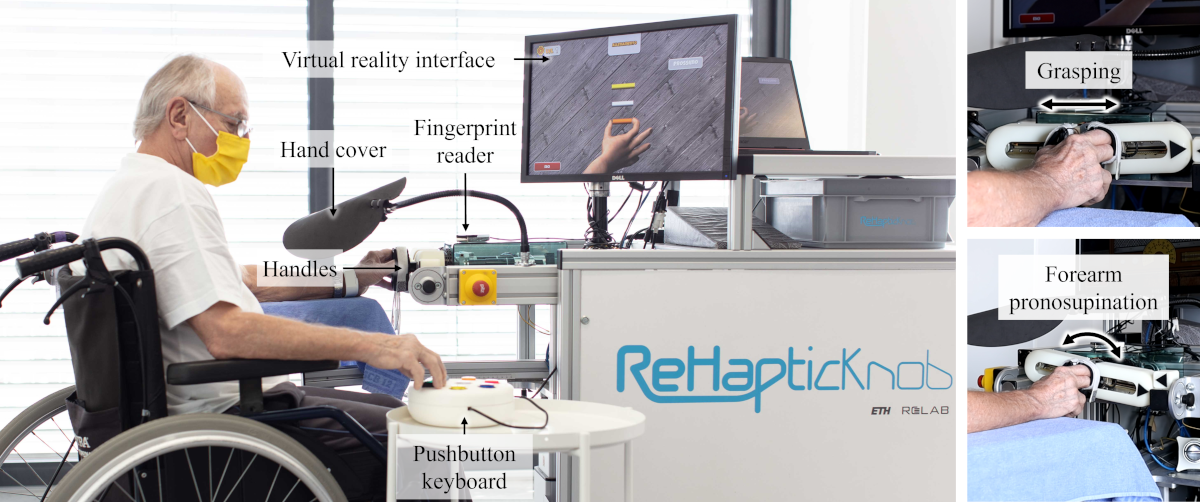
A patient with stroke performing an exercise with the ReHapticKnob. During a therapy session with the device (left), users first log in to their personal therapy account with a fingerprint reader; then, when performing the exercises, they place their fingers on the handles and interact with the virtual reality interface by using a colored pushbutton keyboard. As the exercises implemented on the ReHapticKnob require the user to solve tasks by relying on the sensorimotor information coming from the impaired limb and not on visual information, view of the impaired hand is blocked with a hand cover. The ReHapticKnob allows for grasping (top right) and forearm pronosupination (bottom right) movements.

The rehabilitation technology used in this study is the ReHapticKnob (RHK; Figure 1), which consists of a haptic device for the rehabilitation of hand sensorimotor function after stroke with 2 active degrees of freedom, allowing for hand opening and closing and forearm pronosupination [21].

The rehabilitation exercises implemented on the RHK follow the neurocognitive therapy approach [22-24] and promote motor learning by focusing on sensorimotor integration, as they require patients to use kinesthetic and proprioceptive information from their impaired upper limb and cognitively process it to solve the therapy tasks in the correct way. Assessment-driven exercises ensure an optimal difficulty level from the beginning while ensuring dynamic difficulty adaptation based on the user’s performance [25]. Therapy with the RHK was previously shown to be as efficient as dose-matched conventional therapy [26] when performed in supervised settings.

To make the device suitable for unsupervised use, design changes, such as the implementation of a new graphical user interface and pushbutton keyboard to allow direct interaction between the user and the device, were implemented and tested in a previous usability study [24]. Building on that work, the usability of the platform was further improved by implementing minor graphical changes and audio instructions for the exercises. Furthermore, the platform was improved by integrating clinically inspired algorithms to automatically monitor, control, and adapt therapy in a personalized way [27,28]. This set of algorithms was evaluated in a pilot study with 5 patients with subacute stroke, demonstrating its ability to guide them through several therapy sessions with the RHK and to automatically adapt the difficulty level of the exercises to keep therapy challenging.

### Intervention

**Figure 2:**
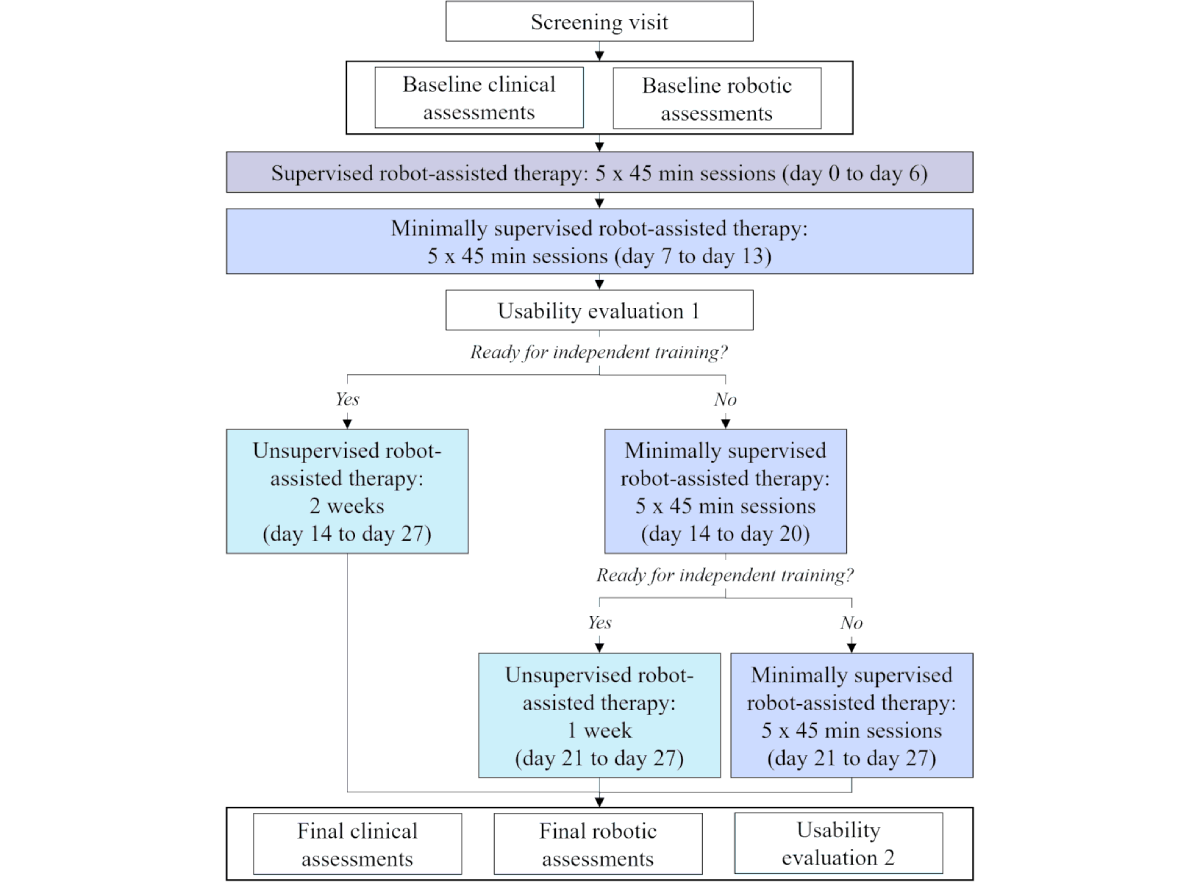
Design of the interventional study for each of the study participants. All participants undergo a screening visit, baseline assessments, 1 week of supervised therapy, and 1 week of minimally supervised therapy. After the first week of minimally supervised therapy, the therapist checks whether the patient properly learned how to use the technology and is ready for unsupervised training. If yes, the patient proceeds to 2 weeks of unsupervised use. If no, an additional week of minimally supervised therapy is performed. At the end of this second week, the therapist checks again whether the patient can train independently. If yes, the patient proceeds to 1 week of unsupervised therapy. If no, an additional week of minimally supervised therapy is performed. In total, each patient performs 4 weeks of technology-assisted rehabilitation. At the end of the protocol, the clinical and robotic assessments as well as the evaluation of the user experience and usability are repeated for all patients.

This trial is a single-group, interventional clinical study. The study protocol lasts for about 4 weeks for each patient (Figure 2), which is compatible with the duration of an inpatient stay at the rehabilitation clinic and comparable to our previous studies with the RHK [26]. The data collected and procedures performed at each time point are listed in Table 1.

**Table 1:** Data collected and procedures (marked with an “X”) performed at each time point of the clinical trial, with primary (PO) and secondary (SO) outcomes indicated. Day 0 corresponds to the first supervised robot-assisted therapy session. The time range indicates how far a procedure can be advanced (–) or postponed (+) for organizational reasons from the precise time point at which it should be performed.

		Screening visit	Baseline clinical assessment	Baseline robotic assessment	Robot-assisted therapy sessions		Usability evaluation 1	Robot-assisted therapy sessions		Final clinical assessment	Final robotic assessment	Usability evaluation 2
Time point		Day –2	Day –1	Day –1	Day 0-6	Day 7-13	Day 13	Day 14-20	Day 21-27	Day 27	Day 27	Day 27
Time range (days)		–4 to +1	±2	±1	—^a^	—	±2	—	—	±2	±2	±2
**Enrolment**												
	Informed consent	X	—	—	—	—	—	—	—	—	—	—
	Eligibility criteria	X	—	—	—	—	—	—	—	—	—	—
	Demographics^b^	X	—	—	—	—	—	—	—	—	—	—
	Medical data^c^	X	—	—	—	—	—	—	—	—	—	—
	Myo-relaxant drugs	X	X	—	—	—	—	—	—	X	—	—
**Assessments**												
	VASp^d^	X	X	—	—	—	—	—	—	X	—	—
	BBT^e^	—	SO	—	—	—	—	—	—	SO	—	—
	MESUPES^f^	—	SO	—	—	—	—	—	—	SO	—	—
	FMA-UE^g^	—	SO	—	—	—	—	—	—	SO	—	—
	mAS^h^	X	SO	—	—	—	—	—	—	SO	—	—
	ABILHAND	—	SO	—	—	—	—	—	—	SO	—	—
	Robotic assessments	—	—	SO	—	—	—	—	—	—	SO	—
	Checklist	—	—	—	—	X	—	(X)^i^	(X)	—	—	—
**Usability**												
	SUS^j^, PSSUQ^k^, TLX^l^, NPS^m^, CSAT^n^	—	—	—	—	—	SO	—	—	—	—	SO
	VAS-smiles^o^	—	—	—	SO	SO	—	SO	SO	—	—	—
**Intervention**												
	Supervised therapy	—	—	—	X	—	—	—	—	—	—	—
	Minimally supervised therapy	—	—	—	—	X	—	(X)	(X)	—	—	—
	Unsupervised therapy	—	—	—	—	—	—	X	X	—	—	—
**Others**												
	Robotic therapy data^p^	—	—	—	PO	PO	—	PO	PO	—	—	—
	Conventional therapy data^q^	—	—	—	X	X	—	X	X	—	—	—
	Adverse events	X	X	X	X	X	X	X	X	X	X	X
	Device deficiencies	—	—	X	X	X	—	X	X	—	X	—

^a^Not applicable.

^b^Demographics include gender, age, year of birth, and hand dominance.

^c^Medical data include stroke onset, lesion type and side, and impaired hand.

^d^VASp: visual analogue scale for pain.

^e^BBT: Box and Blocks Test.

^f^MESUPES: Motor Evaluation Scale for Upper Extremities in Stroke Patients.

^g^FMA-UE: Fugl-Meyer Assessment for upper extremities.

^h^mAS: modified Ashworth Scale.

^i^(X): Performed only for patients who are not ready to train without supervision. These will continue with 5 additional minimally supervised therapy sessions. In this case, the checklist is repeated during the fifth session.

^j^SUS: System Usability Scale.

^k^PSSUQ: Post-Study System Usability Questionnaire.

^l^TLX: raw Task Load Index.

^m^NPS: Net Promoter Score.

^n^CSAT: Customer Satisfaction Score.

^o^VAS-smiles: 5-point visual analogue scale represented by different emoticons used to answer the question “How was your therapy session today?”

^p^Robotic therapy data includes, for example, time of logging in and out, duration of sessions and breaks, number of task repetitions, correct answers, difficulty level of the exercises, and muscle tone data.

^q^Conventional therapy data includes the number and type of standard therapy sessions performed by the patient while participating in the clinical trial.

Before starting the intervention, patients undergo a screening visit with a medical doctor at which the eligibility criteria are checked and demographic and medical data relevant for patient characterization are collected. At the beginning and end of the study, patients undergo a visit with an experienced therapist where clinical assessments are performed to characterize upper limb sensorimotor deficits. Before the first robot-assisted therapy session and at the end of the study, robotic assessments are performed. The baseline robotic assessments are used to tailor the initial difficulty level of the therapy exercises. As robotic therapy is an addition to conventional therapy and this protocol does not comprise a control group, functional recovery is not used to evaluate the efficacy of unsupervised robot-assisted therapy, as it is not possible to infer how much of the recovery is due solely to the addition of robotic therapy, but rather to investigate the possible relation of different impairment profiles on the observed increase in therapy dose.

During the first week of intervention (ie, the supervised phase), a therapist familiarizes the participants with the therapy platform and teaches them how to perform the therapy exercises. During the second week (ie, the minimally supervised phase), participants try to perform the therapy exercises with the device by themselves. A therapist is still present in the room but intervenes only upon request or if deemed to be needed. The first 2 phases consist of 5 sessions of about 45 minutes each, performed on consecutive days (weekends excluded). These sessions are an addition to standard therapy. Therefore, during the study, participants are allowed to fully follow the conventional interdisciplinary rehabilitation program provided by the clinic.

During the last session of the minimally supervised phase, a checklist (Multimedia Appendix 1) is used by the therapist to record whether participants learned how to correctly perform each assigned therapy exercise. The checklist is also used to assess the patient’s functional independence in ambulation and in positioning in front of the device and to decide if the patient meets the requirements to train with the technology in a safe and effective way without supervision. If the requirements are met, the participant can proceed to the unsupervised phase, which lasts for 2 weeks. During the unsupervised phase, patients can independently exercise with the rehabilitation platform during their free time, during dedicated sessions indicated on their therapy schedule, as well as after 5 PM and during the weekend. Specific guidelines on how much to train are not provided. Even though a possible time to train with the device is suggested on the patient’s daily therapy schedule during weekdays, patients are clearly told that they do not necessarily need to attend the session but can rather freely decide when to seek additional therapy within the offered slots or whether to go at all (ie, as a way to simulate home settings).

If, at the end of the first week of minimally supervised therapy, the patients do not meet the requirements to transition to the unsupervised phase, they continue with minimally supervised therapy for an additional week. At the end of the second minimally supervised week, the checklist is performed again. If the patients are now ready for unsupervised training, they can proceed to 1 week of unsupervised therapy; if they are not ready, they undergo a third week of minimally supervised sessions (Figure 2).

During the entire study, the device is placed in an open-access room and an emergency call system is available next to it. Trained personnel of the clinic turn on the device in the morning and turn it off in the evening. If needed, patients are taken to the device by the personnel of the clinic responsible for patient transportation. The personnel might also help patients to position themselves in the correct way in front of the device but do not help with placing the patient’s hand on the handles or operating the device.

The usability of the platform and user experience during robot-assisted therapy is rated a first time at the end of the first week of minimally supervised therapy and a second time at the end of the study. The difference in the ratings between the 2 time points is used to investigate the effect of unsupervised training (ie, of the therapist’s absence) on the perceived usability of the therapy platform and on the user experience during technology-assisted rehabilitation.

Adverse events and device deficiencies are monitored throughout the whole study protocol. The intervention can be discontinued after consultation with medical doctors and therapists or if the participant decides to quit.

For each patient, attendance to the robot-assisted therapy sessions is registered. As attendance to robot-assisted therapy is one of the study outcomes, strategies to increase adherence are not implemented. Reasons for nonattendance are registered a posteriori for each missed supervised or minimally supervised session as well as if patients train for less than 50% of the possible days (ie, less than 7 of 14 days when the patient undergoes 2 weeks of unsupervised therapy or less than 4 of 7 days in cases where the patient undergoes only 1 week of independent training) during the unsupervised phase.

### Outcome Measures

#### Primary Outcomes

The primary outcomes of this study are the feasibility of the proposed protocol, the dose of unsupervised robot-assisted therapy performed by participants, and the difference between usability and user experience between the phases where a therapist is present (ie, supervised and minimally supervised phases) and the unsupervised phase.

Feasibility is measured as the number of patients who could transition to the unsupervised phase out of the total number of tested patients, attendance during the unsupervised phase, and safety of use. Attendance is defined as the percentage of days out of the total number of offered days where the patient trains with the device at least once, while safety is defined as the number of adverse events and device deficiencies throughout the entire protocol.

The different metrics used to characterize therapy dose are therapy duration in minutes, number of task repetitions (ie, the number of target movements performed), and percentage change in physical therapy time with respect to the conventional therapy program. Total conventional physical therapy time is estimated from the sum of the duration in minutes of the physiotherapy (without differentiating between upper limb and lower limb) and occupational therapy sessions regularly performed by each patient at the clinic.

Platform usability is assessed with the System Usability Scale [29], the raw Task Load Index [30], and the Post-Study System Usability Questionnaire [31]. User experience during robot-assisted therapy is assessed with the Net Promoter Score [32] and the Customer Satisfaction Score, as well as by evaluating the reasons (if any) for not attending the therapy sessions with the device.

The results of the checklist used to define which patient can proceed to unsupervised therapy are also used to identify specific aspects of the platform that might need to be further improved.

#### Secondary Outcomes

The difference in intensity (ie, number of task repetitions per minute) and task performance during supervised or minimally supervised therapy and unsupervised therapy is used to evaluate if the content of the therapy varies when the therapist is no longer present. A task repetition corresponds to the target action that the patient must perform in a given exercise (eg, a movement or combination of movements, such as one opening and closing of the hand or interacting with one virtual object rendered by the robot). A third metric to evaluate the difference in therapy content is the ratio of effective therapy time (ie, net therapy time without breaks) to total duration of a therapy session during supervised or minimally supervised therapy and unsupervised therapy.

Functional recovery, assessed as the difference between the baseline and final scores in the clinical and robotic assessments, is another secondary outcome. Clinical assessments comprise the Fugl-Meyer Assessment for upper extremities [33], ABILHAND [34], the Box and Block Test [35], the Motor Evaluation Scale for Upper Extremities in Stroke Patients [36], and the modified Ashworth Scale [20]. Robotic assessments comprise active range of motion, hand proprioception, and haptic perception (A1-A3 in [25]).

The scores of the cognitive assessments performed during the screening visit (Goodglass-Kaplan scale and levels of cognitive functioning-revised) are used to gain more insights into the effect of cognitive function on the ability to transition to the unsupervised phase.

Other parameters possibly influencing therapy dose or attendance are also evaluated. These comprise age, baseline assessment scores, functional recovery, the number of total conventional therapy sessions, and functional independence and mobility, as assessed with the Barthel index [37] and custom questions every time the checklist is performed (ie, at the end of each week of minimally supervised therapy).

### Sample Size

The study includes 13 patients; 10 patients are expected to be sufficient to perform a feasibility study and gather information on the potential of unsupervised therapy to plan a subsequent larger study targeting efficacy, and a dropout rate of around 20% is accounted for based on our previous studies [26]. The selected sample size guarantees the execution of the study within a relatively short time (ie, 2 years) such that improvements to the device, if needed, can be implemented in a timely manner for follow-up studies. Furthermore, this sample size is comparable to previous studies performed by our group with the RHK and to other studies with similar goals [10,11,15].

### Data Collection, Management, and Confidentiality

All the data listed herein are collected by trained personnel. For withdrawn participants, the clinical and robotic data collected until withdrawal are kept, and no further clinical or robotic evaluations are performed after that. A specific follow-up phase is not needed.

At the beginning of the study an ID is assigned to each patient and the saved data are coded according to this ID. Study data are reported on a paper case report form (CRF), 1 for each patient, and then entered in an electronic CRF with the help of an electronic database, namely Research Electronic Data Capture (REDCap) [38,39], hosted on a secure server at the Swiss Federal Institute of Technology Zürich. REDCap promotes data quality via functions and settings, like audit trails, field type restriction, range checking, and definitions of required fields. Data automatically saved by the RHK are stored on the connected laptop. Backups of the data saved on the laptop and of the electronic CRF are saved monthly on the protected server of the sponsor institution. To promote security, the laptop is password protected and participants can only access the therapy interface. The data will be archived for a minimum of 10 years after study termination or premature termination of the clinical trial.

### Data Analysis

Descriptive statistics will be used to describe the study population (eg, demographics, initial clinical scores, and changes in clinical scores, such as functional recovery), attendance and therapy dose in the unsupervised phase, user experience, and platform usability.

Normal distribution of the data will be checked with the Shapiro Wilk test. For each usability metric, a paired *t* test (normal distribution) or a paired samples Wilcoxon test (for data not normally distributed) will be performed to compare the data collected during the usability evaluation 1 and usability evaluation 2 sessions.

To evaluate the content of unsupervised therapy, 1-way repeated measures ANOVA or its nonparametric version (Friedman test) with post hoc tests will be run to compare intensity (average block intensity for each patient), performance, and ratio of effective therapy time to total session duration during supervised, minimally supervised, and unsupervised therapy. The same tests will be used to compare the answers to the VAS-smiles, a 5-point VAS represented by different emoticons, performed at the end of each therapy session during the 3 phases. For these comparisons, we expect therapy content and VAS-smiles not to vary significantly depending on the level of supervision, as ideally the absence of the therapist should not impact these outcomes. Patients who did not reach the unsupervised phase will be excluded from this analysis.

Linear mixed-effects models will be used to investigate parameters (eg, demographics and baseline data) possibly influencing the achieved dose of unsupervised therapy.

Relevant qualitative data will be summarized and reported accordingly.

Data analysis will be performed with MATLAB R2021b (MathWorks).

### Monitoring

Monitoring is performed by a study nurse employed at the sponsor institution. An initiation visit on-site is performed to check the suitability of the infrastructure and staff. Routine monitoring is then performed through on-site or web-based visits depending on the needs of the investigators (eg, on request or for safety reasons). A close-out visit is performed after the completion of the study. Interim analysis is not foreseen.

Observed or spontaneously reported adverse events are collected and assessed by one of the medical doctors involved in the clinical study and recorded on the CRF. Adverse events are then reported in the annual safety report and final report. Serious adverse events are reported to the ethics committee and to the competent authority within 7 days.

Auditing may be conducted at any time by the ethics committee or the competent authority, both independent from the sponsor and investigator. The study documentation, CRF, and informed consent are accessible for auditing.

### Ethical Considerations

This study protocol follows the SPIRIT (Standard Protocol Items: Recommendations for Interventional Trials) reporting guidelines [40]. The study follows the guidelines on good clinical practice and the Declaration of Helsinki. The protocol (Version 2, 17.03.2020) has been approved by the cantonal ethics committee (“Comitato etico cantonale Ticino,” CE TI 3577) and by the Swiss Agency for Therapeutic Products (Swissmedic, 102681300) and was registered at ClinicalTrials.gov (NCT04388891) and on the portal for clinical trials in Switzerland (SNCTP000003850). Substantial amendments have to be submitted and approved by the cantonal ethics committee and Swissmedic before being implemented and distributed to the study staff both orally and in writing.

Ancillary and post-trial care is not needed. An insurance was stipulated to compensate for eventual harm from trial participation.

The sponsor and investigators will have access to the final trial data set. The study results will be presented among the collaborators of the Clinica Hildebrand Centro di Riabilitazione Brissago and of the sponsor institution. Results are planned to be published in peer-reviewed journals or conferences. The use of a professional writer and public access to the data set and statistical code is not foreseen.

## Results

Recruitment started in November 2020. As of April 2023, 13 patients were recruited and completed the protocol. Data analysis is ongoing, and publication of the results is expected for 2023.

## Discussion

This paper describes the details of a single-group, interventional clinical study investigating the feasibility of unsupervised robot-assisted therapy of hand function in inpatients with subacute stroke and its effect on the dose of therapy. In particular, we propose a systematic approach for a progressive transition from supervised to unsupervised use of an advanced rehabilitation technology.

Unsupervised technology-assisted rehabilitation is seen as a promising avenue to increase therapy dose for stroke survivors without increasing the workload for therapists. As opposed to most telerehabilitation interventions, whose development was fueled, for instance, by the COVID-19 pandemic and the need for neurorehabilitation services to decrease reliance on hospital presence (eg, by proposing remotely supervised physiotherapy sessions [41,42]), robot-assisted therapy may not rely on the presence of a therapist [7]. Such a fully unsupervised approach to neurorehabilitation might therefore provide a more sustainable answer to the unfavorable patient to therapist ratio [4,43].

The absence of supervision during rehabilitation certainly raises challenges with respect to adherence and motivation to train, understanding of tasks to be achieved, and safety. We expect that an initial standardized training procedure can help patients to learn how to use a rehabilitation technology in a controlled way so that the risk of adverse events during unsupervised therapy is reduced and patients feel comfortable and motivated in using the technology on their own. Despite the many feasibility studies describing unsupervised technology-assisted rehabilitation, there exists no consensus on how to optimally achieve the transition from supervised to unsupervised use of such technologies, nor is there an agreement on how to define and evaluate feasibility. The systematic approach outlined in this protocol paper provides a way for patients to progressively transition from supervised to unsupervised use of a rehabilitation technology based on objective data (ie, a checklist). Furthermore, it allows for the collection of information on the feasibility of unsupervised therapy and its effect on therapy dose, user experience, and the usability of the rehabilitation technology used. We expect this systematic approach to allow patients to transition to unsupervised use of a rehabilitation technology in a safe and comfortable way, meaning that usability and user experience do not drop during unsupervised use. High perceived usability and user experience are essential and increasingly investigated aspects [11,24,44] as they may increase adherence, and therefore, therapy dose, in the unsupervised phase, potentially leading to an improvement in functional outcomes.

While designed here for a specific robotic technology (ReHapticKnob), the proposed methodological approach could be generalized to other rehabilitation technologies with minor adjustments, such as the goals reported in the checklist, which need to be adapted to the specific rehabilitation platform as they depend on the practical actions that users need to learn to use a given technology. Furthermore, depending on the complexity of the technology, the number of supervised and minimally supervised sessions might be adapted. All other outcome measures, such as the usability and user experience questionnaires, adverse events, device deficiencies, and clinical assessments, can be collected independently from the technology used. Outcomes used to characterize therapy dose, namely, therapy duration in minutes or the number of repetitions, should be parameters that most rehabilitation technologies automatically record, as one or both of them are already reported by many research groups working in the field [13,45-47]. Given its generalizability, the proposed methodological approach could serve as a benchmark to allow for better and easier comparisons between different technologies for unsupervised rehabilitation.

The proposed interventional study of patients with subacute stroke aims to demonstrate the feasibility of fully unsupervised robot-assisted rehabilitation in a clinical setting. While no supervision is provided during the use of the device in the unsupervised phase of the study, the fact that it takes place in a clinical environment may still influence adherence and the achieved dose. However, if successful in terms of feasibility, the approach outlined in this paper could, as a next step, also be applied to the home setting. For example, the supervised and minimally supervised (and even the unsupervised) phases can be implemented in the clinic, and after discharge, the patient could continue training with the technology in an unsupervised way at home. The purpose of the progressive transition approach we propose here would be to guarantee that enough training in the use of the device is provided before discharge to later allow for safe and high-quality unsupervised use of the robot at home, which could help keep patients engaged in rehabilitation and maintain or even further increase functional recovery.

## Appendix

Multimedia Appendix 1 Checklist used by the therapist to record whether participants learned how to correctly perform each assigned therapy exercise, the patient's functional independence in ambulation and in positioning in front of the device, and to decide if the patient meets the requirements to train with the technology in a safe and effective way without supervision.

Multimedia Appendix 2 Checklist for SPIRIT (Standard Protocol Items: Recommendations for Interventional Trials) guidelines.

## Abbreviations

CRF: case report form
REDCap: Research Electronic Data Capture
RHK: ReHapticKnob
SPIRIT: Standard Protocol Items: Recommendations for Interventional Trials
VAS: visual analogue scale

## Data Availability

This paper describes a research protocol and does not report collected data. The data sets generated during the study detailed here will later be presented in the related main manuscript describing the results of the study and will be available from the corresponding author upon reasonable request.
