# Supplementary material for: Progressive Transition From Supervised to Unsupervised Robot-Assisted Therapy After Stroke: Protocol for a Single-Group, Interventional Feasibility Study
Source: JMIR Res Protoc. 2023 Nov 9;12:e48485. doi: 10.2196/48485 (PMC10667973; doi:10.2196/48485)
Supplement: Multimedia Appendix 1 [file resprot_v12i1e48485_app1.pdf]

Checklist

This is a Multimedia Appendix to a full manuscript published in JMIR Research Protocols. For full copyright and citation information see "Devittori G., Ranzani R., Dinacci D., Romiti D., Califfi A., Petrillo C., Rossi P., Gassert R. and Lambercy O. *Progressive Transition From Supervised to Unsupervised Robot-Assisted Therapy After Stroke: Protocol for a Single-Group, Interventional Feasibility Study*".

This checklist generally matches the one used in the study (redundant information has been shortened), but instructions for use and footnotes have been added to allow other research groups to use it.

GENERAL USE OF THE DEVICE[1]

**Goal**	**Notes with date (e.g.: difficulties present at the end of the week, what creates problems, reason)**	**End of FIRST week of minimally supervised therapy**
The patient is able to correctly position the hand on the handles	__/__/__ : __________<br>__________<br>__/__/__ : __________<br>__________	Yes ☐ No ☐
The patient is able to access his/her personal therapy account with the fingerprint	__/__/__ : __________<br>__________<br>__/__/__ : __________<br>__________	Yes ☐ No ☐
The patient is able to leave/quit the therapy session when desired ("Quit")	__/__/__ : __________<br>__________<br>__/__/__ : __________<br>__________	Yes ☐ No ☐
The patient completed the therapy sessions without needing to seek external help for relevant reasons	__/__/__ : __________<br>__________<br>__/__/__ : __________<br>__________	Yes ☐ No ☐
The patient is able to correctly perform at least one exercise with the ReHapticKnob	__/__/__ : __________<br>__________<br>__/__/__ : __________<br>__________	Yes ☐ No ☐

[1] These goals are specific for the ReHapticKnob but can be adapted to meet the requirements of different rehabilitation technologies.

**EXERCISE SPECIFIC PART**[2]				
**Exercise**		**Goals (with potential notes)**	**End of FIRST week of minimally supervised therapy**	
			**Yes**	**No**
**Bars**	Executed?[3] Yes ☐ No ☐	The patient is able to place the hand horizontally and select the position with the green button (calibration).	☐	☐
		The patient is able to close the handles as far as possible and select the position with the green button (calibration).	☐	☐
		The patient is able to start the therapy exercise by pressing the green button ('Start').	☐	☐
		The patient is able to grasp or touch objects or interact correctly with the therapy exercise.	☐	☐
		After the break, when the patient feels ready, he/she is able to restart the therapy exercise by pressing the green button.	☐	☐
		The patient is capable of quitting the therapy exercise at any time.	☐	☐
		____		
		____		
Exercise x[4]	Executed? Yes ☐ No ☐	*Goal 1: Define here a goal specific for this exercise*	☐	☐
		Goal …	☐	☐
		____		
		____		

EVALUATION - End of FIRST week of minimally supervised therapy

- In the section **"General use of the device" all answers are "Yes"**. The patient can then move on to the unsupervised therapy phase.

  Yes ☐ **No** ☐ ⇒ **If "No"**: Continue with minimally-supervised therapy and then complete the "*If applicable: End of SECOND week of minimally-supervised therapy*" section.

- Section "**Exercise specific part**"*:* list the executed exercises where the patient scored one or more "No" and which will therefore be excluded from his/her therapy plan:

  ____

Barthel Index – Mobility - End of FIRST week of minimally supervised therapy

0	Immobile
5	Wheelchair independent for >45 meters
10	Need the help of one person for > 45 meters
15	Independent for > 45 meters (may use any aid, e.g., stick, but not a walker)

Further information - At the end of the first week of minimally supervised therapy the patient:

☐ moves safely on his own ☐ must be accompanied to the sessions with the ReHapticKnob

☐ walks without help

moves with the help of: ☐ crutches ☐ walker ☐ wheelchair

☐ needs help in positioning in front of the device: specify

____

____

[2] For each exercise available for the investigated rehabilitation technology, a list of goals that the patient has to reach in order to be able to correctly perform the exercise has to be defined. Some goals can vary depending on the exercise, while others can stay the same. An example is provided for the exercise "Bars" implemented on the ReHapticKnob.

[3] This option allows you to define whether the exercise has been assigned to the patient (and thus included in his or her therapy plan) or not, as not all the exercises implemented on a rehabilitation technology are suitable for all the patients.

[4] The original checklist had a set of goals for each of the 7 exercises implemented on the ReHapticKnob. Here it was shortened for illustration purposes.

To be completed ONLY if the patient cannot progress to the unsupervised phase after the FIRST or eventually after the SECOND week of minimally supervised therapy.

GENERAL USE OF THE DEVICE			
Goal	Notes with date (e.g.: difficulties present at the end of the week, what creates problems, reason)	If applicable: End of SECOND week of minimally supervised therapy	If applicable: End of THIRD week of minimally supervised therapy
The patient is able to correctly position the hand on the handles	__/__/__ : ____<br>__/__/__ : ____<br>__/__/__ : ____	Yes ☐ No ☐	Yes ☐ No ☐
The patient is able to access his/her personal therapy account with the fingerprint	__/__/__ : ____<br>__/__/__ : ____<br>__/__/__ : ____	Yes ☐ No ☐	Yes ☐ No ☐
The patient is able to leave/quit the therapy session when desired ("Quit")	__/__/__ : ____<br>__/__/__ : ____<br>__/__/__ : ____	Yes ☐ No ☐	Yes ☐ No ☐
The patient completed the therapy sessions without needing to seek external help for relevant reasons	__/__/__ : ____<br>__/__/__ : ____<br>__/__/__ : ____	Yes ☐ No ☐	Yes ☐ No ☐
The patient is able to correctly perform at least one exercise with the ReHapticKnob	__/__/__ : ____<br>__/__/__ : ____<br>__/__/__ : ____	Yes ☐ No ☐	Yes ☐ No ☐

EXERCISE SPECIFIC PART						
**Exercise**	**Goals (with potential notes)**	**If applicable: End of SECOND week of minimally supervised therapy**		**If applicable: End of THIRD week of minimally supervised therapy**		
		**Yes**	**No**	**Yes**	**No**
**Bars** Executed? Yes ☐ No ☐	The patient is able to place the hand horizontally and select the position with the green button (calibration).	☐	☐	☐	☐
	The patient is able to close the handles as far as possible and select the position with the green button (calibration).	☐	☐	☐	☐
	The patient is able to start the therapy exercise by pressing the green button ('Start').	☐	☐	☐	☐
	The patient is able to grasp or touch objects or interact correctly with the therapy exercise.	☐	☐	☐	☐
	After the break, when the patient feels ready, he/she is able to restart the therapy exercise by pressing the green button.	☐	☐	☐	☐
	The patient is capable of quitting the therapy exercise at any time.	☐	☐	☐	☐
	____ ____				
Exercise x Executed? Yes ☐ No ☐	*Goal 1: Define here a goal specific for this exercise*	☐	☐	☐	☐
	Goal…	☐	☐	☐	☐
	____ ____				

EVALUATION - End of SECOND week of minimally supervised therapy

- In the section **"General use of the device" all answers are "Yes"**. The patient can then move on to the unsupervised therapy phase.

  Yes ☐ **No** ☐ ⇒ **If "No"**: Continue with minimally-supervised therapy and then complete the "*If applicable: End of THIRD week of minimally-supervised therapy*" section.

- Section "**Exercise specific part**"*:* list the executed exercises where the patient scored one or more "No" and which will therefore be excluded from his/her therapy plan:

  ____

Barthel Index – Mobility - End of SECOND week of minimally supervised therapy

0	Immobile
5	Wheelchair independent for >45 meters
10	Need the help of one person for > 45 meters
15	Independent for > 45 meters (may use any aid, e.g., stick, but not a walker)

Further information - At the end of the SECOND week of minimally supervised therapy the patient:

☐ moves safely on his own ☐ must be accompanied to the sessions with the ReHapticKnob

☐ walks without help

moves with the help of: ☐ crutches ☐ walker ☐ wheelchair

☐ needs help in positioning in front of the device: specify

____

____
